# What are the barriers, facilitators and interventions targeting help-seeking behaviours for common mental health problems in adolescents? A systematic review

**DOI:** 10.1186/s12888-020-02659-0

**Published:** 2020-06-11

**Authors:** Antonia Aguirre Velasco, Ignacio Silva Santa Cruz, Jo Billings, Magdalena Jimenez, Sarah Rowe

**Affiliations:** 1 Child and Adolescent Mental Health Service, Children’s Hospital Dr. Roberto del Río, Santiago, Chile; 2 Public Health School, Faculty of Medicine, University of Chile, Santiago, Chile; 3 División of Psychiatry, University College London, London, UK; 4 Camden and Islington Mental Health Foundation Trust, London, UK

**Keywords:** Help-seeking, Adolescent, Mental health, Barriers, Facilitators, Interventions, Systematic review

## Abstract

**Background:**

Increasing rates of mental health problems among adolescents are of concern. Teens who are most in need of mental health attention are reluctant to seek help. A better understanding of the help-seeking in this population is needed to overcome this gap.

**Methods:**

Five databases were searched to identify the principal barriers, facilitators and interventions targeting help-seeking for common mental health problems in adolescents aged 10–19 years. The search was performed in June 2018 and updated in April 2019. Two independent screening processes were made using the eligibility criteria. Quality assessment of each study was performed, and findings summarised using a narrative synthesis.

**Results:**

Ninety studies meet the inclusion criteria for this review for barrier and facilitators (*n* = 54) and interventions (*n* = 36). Stigma and negative beliefs towards mental health services and professionals were the most cited barriers. Facilitators included previous positive experience with health services and mental health literacy. Most interventions were based on psychoeducation, which focused on general mental health knowledge, suicide and self-harm, stigma and depression. Other types of interventions included the use of multimedia and online tools, peer training and outreach initiatives. Overall, the quality of studies was low to medium and there was no general agreement regarding help-seeking definition and measurements.

**Conclusion:**

Most of the interventions took place in an educational setting however, it is important to consider adolescents outside the educational system. Encouraging help-seeking should come with the increased availability of mental health support for all adolescents in need, but this is still a major challenge for Child and Adolescent Mental Health Services. There is also a need to develop shared definitions, theoretical frameworks and higher methodological standards in research regarding help-seeking behaviours in adolescents. This will allow more consistency and generalisability of findings, improving the development of help-seeking interventions and ensuring timely access to mental health treatments.

## Background

Young people present with the highest prevalence of mental health disorders compared to individuals at any other stage of the lifecycle [1], with up to 20% of adolescents likely to experience mental health disorders [2]. Mental health has been defined as “a state of wellbeing in which and individual realizes of his/her abilities, can cope with normal stresses of life ( …) and is able to make a contribution to his/her community” [3]. Around 50% of mental health conditions start before the age of 14 [4] and the onset of 75% of cases is before the age of 18 [5]. The most common diagnoses are depression and anxiety [6] and around 25% of young people experience psychological distress [7]. Depression is one of the principal causes of illness and disability in teenagers, and suicide is the third most common cause of death among older adolescents [4]. Mental health problems can significantly affect the development of children and young people [4] having an enduring impact on their health and social functioning in adulthood [8]. Adolescents experiencing mental health conditions may face several challenges such as isolation, stigma, discrimination and difficulty in accessing health services [2]. However, 75% of adolescents with mental health problems are not in contact with mental health services [9], the primary reason being reluctance to seek help [1, 10, 11].

Help-seeking for mental health problems necessitates communicating the need for personal and psychological assistance to obtain advice and support. Rickwood and Thomas’ (2012) define help-seeking for mental health problems as “an adaptive coping process that is the attempt to obtain external assistance to deal with mental health concerns” [p.180, 12]. This includes both formal (e.g., health services) and informal (e.g., friends and family) sources of help. However, adolescents most in need of psychological help are those least likely to look for it [1–13]. One of the biggest challenges in adolescent mental health is ensuring that at-risk individuals are linked with the appropriate support [14]. Understanding barriers and facilitators to help- seeking is fundamental for the development of interventions and programmes to support adolescents with mental health problems.

Rickwood et al., (2005), investigated the main barriers and facilitators of help-seeking for mental health problems in young people. They found that lack of emotional competence, negative beliefs about help-seeking and stigma were the most prominent barriers. Conversely, emotional competence, previous positive experiences with health professionals and mental health literacy, were the main facilitators [15]. Gulliver et al., (2010) performed a systematic review of the available literature at that time, finding similar results; however, they stated that stigma was the most prominent barrier for seeking for help in young people [1]. Another systematic review was made by Rowe et al., (2014), focused on in help-seeking for adolescent self-harm. They found that in addition to stigma, negative reactions from others related to confidentiality breaches and being seen as an “attention seeker” were the most relevant obstacles [10]. While interesting, these previous reviews do not address the help-seeking barriers and facilitators of most common mental health troubles among adolescents, nor include interventions targeting these. Rickwood, Deane et al., (2005) only included depressive symptoms, personal emotional problems and suicidal thoughts and Rowe et al. (2014), only focused on adolescent self-harm. The most complete review published by Gulliver and colleagues (2010) is almost 10 years old and need of updating.

Adequate and effective interventions that promote help-seeking are necessary for enhancing prevention, early detection, timely treatment and recovery from mental health problems [14]. Previous systematic reviews on interventions targeting help-seeking reveal some promising results in regard to enhancing mental health literacy [16] and a significant positive overall effect of these interventions in improving help-seeking for mental health problems [17]. Nonetheless, these reviews do not focus on adolescent populations and only one includes randomised controlled trials (RCT).

The primary aim of this review is therefore to provide an update of the literature on barriers and facilitators of adolescent mental health help-seeking including formal and informal sources of help, with the inclusion of interventions targeted at improving this. We will focus on common mental health problems, including depression, anxiety, suicidal thoughts, self-harm, emotional distress, among other personal-emotional symptoms. The secondary outcome is to examine any significant differences between age and sex. Understanding the difficulties around help-seeking behaviours and facilitating access to timely and effective treatment is essential for preventing the escalation of mental health problems among adolescents.

## Methods

For the purpose of this review, help-seeking was defined as the action of actively searching for help for mental health problems, including informal (family, friends) or formal (GP, mental health professionals, etc.) sources, based on interpersonal and social abilities [11]. “Adolescents” were people aged 10 to 19 years, as defined by the World Health Organisation [4]. Despite the increasing debate regarding the age of adolescence [18], this definition was considered as appropriate for our study as it is accepted by international organisation such as OMS and UNICEF. Also, we considered this age range more homogenous and comparable in terms of lifecycle experiences and challenges that would be reflected in help-seeking behaviours and intentions. This review was prospectively registered on PROSPERO (CRD42018096917) and reported in accordance with the PRISMA guidelines [19]. The search terms were developed using the PICO structure, then expanded using MeSH terms and combined using Boolean operators. Four databases were selected including MEDLINE®, Embase, PsycINFO, and Web of Science, as well as the search engine Google scholar, identified as an optimal database combination [20]. Grey literature from the mentioned databases was also included and a search was carried in Open Grey. An initial version of the proposal for this study was reviewed by the McPin Foundation. The feedback was considered in the developmental stage, in order to evaluate the relevance and reception of the protocol by Patient and Public Involvement (PPI) organisations.

We included studies published in English, Spanish and French and focused on identifying barriers, facilitators and interventions targeting help-seeking behaviours for mental health problems in adolescents, specifically depression, anxiety, suicidal ideation, emotional distress and general symptoms of mental illness. Other mental health problems such as psychosis, anorexia, among others were excluded, because we decided to focus on most prevalent mental health problems which share a more similar help-seeking process. Regarding barriers and facilitators, we included studies published after 2010 since a previous systematic review on the topic was published then [1]. We did not include any limit regarding year of publication for help-seeking interventions. All study designs were considered, including feasibility studies and study protocols. We excluded studies that referred to young people over the age of 19 or children under 10 years old. When study populations included adolescents outside of the established age range, the paper was included if over 50% of the individuals in the sample were within the 10–19 years category or if separate outcome data was provided for the participants in this age range. Studies meeting the inclusion criteria and including parents in their sample were also considered. Finally, other exclusion criteria were articles written in other languages, or if the intervention did not explicitly target help-seeking behaviours or was not related to mental health conditions (Appendix S1) (Table 1).

**Table 1:** Inclusion/Exlusion criteria

Inclusion	Exclusion
- Focused on help-seeking barriers, facilitators and interventions - Includes formal and informal sources of help - Help-seeking for depression, anxiety, suicidal ideation, emotional distress - Age range: 10 to 19 years - Studies published in English Spanish and French - All study designs	- Not focused on help-seeking for mental health problems - Help-seeking for anorexia, schizophrenia and other mental health problems not described in the inclusion criteria - Participants above or over 10 to 19 years age range - Published in other languages - Published before 2010 if referring to help-seeking barriers and facilitators

The search was performed in June 2018 and updated in April 2019. The results were exported to EndNote X8 and duplicates were removed. Titles and abstracts were screened by one author (AA) at the first stage. At a second stage, two authors (AA and IS) checked the full articles using the pre-determined inclusion and exclusion criteria. A third member of the research team (MJ) was available to solve discrepancies. Disagreement on 12 studies was attributed mainly to differences concerning the definition and measurement of help-seeking and was resolved in a discussion with a third author (MJ) not involved in the process of screening. Authors were contacted when relevant information was missing or when we could not find the articles retrieved by the databases. Reference list of all included studies were screened in case we found other studies relevant to our review. Data were extracted using a predefined form, which allowed the research team to identify the main characteristics of each study. This process was executed by one author (AA) after a complete review of the included papers. For the first question, data extraction focused on identifying barriers and facilitators and for the second question, intervention and effect size when reported. We created an additional form to extract data regarding the secondary outcome (age and sex). For the quality assessment, we used the Joanna Briggs Institute Critical Appraisal Checklist [21] and the Mixed Methods Appraisal Tool (MMAT) [22], which were appropriate due to the variety of study designs included in this review; both have been previously validated [23, 24]. The Joanna Briggs tool has a number of checklists to evaluate the main features of each study design. We used the checklist for cross-sectional studies, RCT, quasi-experimental studies and qualitative studies. Each checklist had a number of items to evaluate the most relevant aspect of the specific design (e.g: for RCT was allocation to treatment groups concealed? Were treatment groups similar at the baseline?). After completing the checklist an overall quality appraisal score was calculated to provide a measure (low, medium and high) of the quality of each study. The MMAT included a similar checklist but is specific to mixed method study reviews. Overall study quality was not used as an exclusion criterion because we opted to be overly inclusive and provide a thorough overview of help-seeking in adolescents. Results have been summarised using a narrative synthesis. We identified the most relevant features regarding help-seeking barriers, facilitators and interventions in our data. These features were grouped into themes that capture the essential aspects regarding the main outcome of this review. With this information we developed a preliminary synthesis of the results organizing the themes so that patterns regarding the main barriers, facilitators and interventions were identified. Finally, we explored previous evidence on the topic and explore the relationship between the included studies. This allowed us to explore the influence of heterogeneity and the robustness of the preliminary synthesis [25]. Due to the heterogeneous nature of the studies included, a meta-analysis was not conducted. The quality of each study was not used as an exclusion criterion or impacted the weight given to each study in the narrative synthesis. This over-inclusive criteria allowed us to have an overall picture not only regarding help-seeking barriers, facilitators and interventions, but also the methodological quality of the available evidence.

## Results

**Fig. 1:**
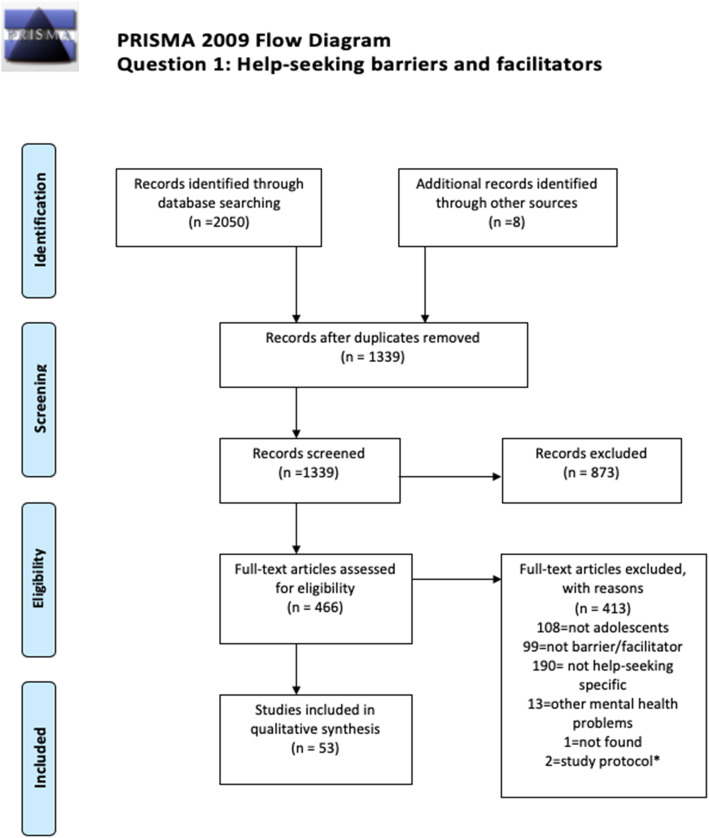
Prisma 2009 Flow Diagram. Question 1: Help-seeking barriers and facilitators

**Fig. 2:**
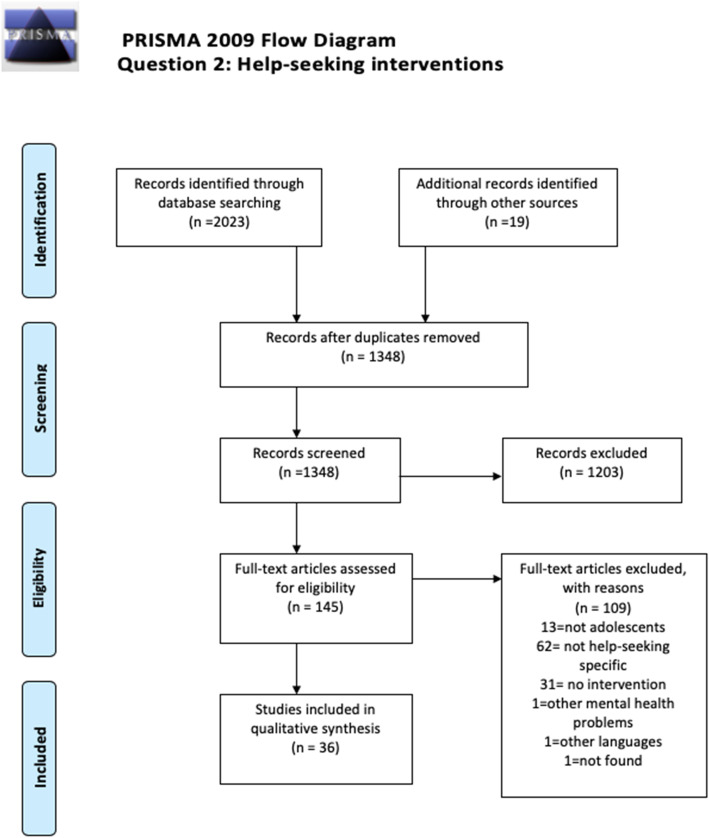
Prisma 2009 Flow Diagram. Question 2: Help seeking interventions

Two independent searches were carried out during June 2018 and then updated in April 2019. A total of 90 studies were included in this review, combining both barriers and facilitators (*n* = 54) and the intervention (*n* = 36) questions. PRISMA diagrams displaying the number of papers retrieved and the process of selection of the included studies is available in Figs. 1 and 2. Regarding the inter-rater reliability for this review, the agreement between the researchers screening the papers was high, with a 85% accuracy and 95% precision (Kappa = 0.954). Adolescents identified a range of formal and informal help-seeking options across studies, such as GPS, psychologists, psychiatrists, teachers, social workers (formal), and friends, family, sporting coaches, and online communities (informal). Regarding question 1, most of studies focused on identifying barriers and facilitators towards formal sources of help, whereas intervention studies had a wider variety of sources of help, depending on help-seeking behavior attempted to promote.

### Question 1: help-seeking barriers and facilitators

Fifty-four studies that reported barriers and/or facilitators including a total of 56,821 participants were considered in the narrative synthesis (Table 2). Most of the studies (*n* = 18) were conducted in Australia, followed by the United States (*n* = 12) and the United Kingdom (*n* = 5). The majority of the studies were cross sectional (*n* = 36) [26–61], thirteen studies were qualitative [62–74] and six used a mixed-method design [75–80]. Three PhD dissertations and one conference abstract were included in the grey literature. The age ranged from 8 to 26 years old. Three articles included adolescents and their parents, while one article included just adolescents’ mothers.

**Table 2:** Barriers and facilitators

1st author, year, country	Study design	Sample size (n=)	Age and setting	Barriers	Facilitators	Quality
Bates, 2012, Canada	Cross sectional survey	*n* = 193 students 110 parents	11 to 15 years old high school students	Students: “nothing will help”, stigma, self-sufficiency, fear of coercion. Parents: fear of coercion, money constraints, self-sufficiency, perceived impact of adolescent problems and not understanding the child problem.	Both: Prior professional help-seeking	Medium
Boyd, 2011, Australia	Cross sectional	*n* = 201	11 to 18 years old students in rural high school	Perceived limited service availability, social proximity and gossip, travel and cost of service, limited knowledge of sources of help, fears confidentiality	Not assessed	Medium
Buttigieg, 2016, Malta	Mixed Methods	*n* = 494	14–15-year-old high school students	Need for autonomy, embarrassment, poor mental health literacy, stigma, higher levels of depressive symptoms	Not assessed	Low
Charman, 2010, Australia	Qualitative	n = 20	16–26 (mean 17.7 years) members of community groups	Confidentiality concerns and stigma	Not assessed	Medium
Chen, 2017, Malasya	Cross sectional	*n* = 277	13–20-year-old high school students	Stigma, fear, lack of courage, doubt about counsellor competency	Not assessed	Medium
Cheng, 2013, United Kingdom	Cross sectional	*n* = 67	Parents of Chinese students in language school living in England.	Knowledge about help-seeking, language barriers	Not assessed	Low
Cramer, 2017, United States	Cross sectional	*n* = 396	14–17-year-old high school students	Stigma, higher levels of emotional difficulties, personnel and service availability	Prior help-seeking behaviours	Medium
Curtis, 2010, New Zealand	Mixed Methods	*n* = 1896	18–24 years old (60.2% under 20) university students	Stigma and a perceived need for self-reliance	Not assessed	Low
Czyz, 2013, United States	Cross sectional	*n* = 157	18–22-year-old (77.1% under 20) college students at elevated suicide risk	Perception that treatment is no needed, lack of time, self-management and stigma	Not assessed	Low
Dardas, 2017, Jordania	Cross sectional	*N* = 2349	12–17 year-old high school students	Higher depressive symptoms, higher levels of stigma	Not assessed	Medium
De Anstiss, 2010, Australia	Qualitative	*n* = 85	13–17 years old, refugee adolescents living in Australia	Low priority of mental health, poor mental health literacy, distrust in services, stigma associated with psychological problems and help-seeking	Not assessed	Medium
Doyle, 2017, Ireland	Mixed methods	*n* = 856	15–17-year-old students in post-primary school	Dislike of dual role of counsellor/teacher, confidentiality concerns.	Not assessed	Medium
Fukuda, 2016, Brazil	Cross sectional	*n* = 1030	8–21-year-old school students receiving mental health treatment	Fear of stigmatisation and problem denial	Not assessed	Low
Flink, 2013a, The Netherlands	Qualitative	*n* = 41	Mother of teen daughters (aged 12–20) from different ethnic backgrounds	Negative attitudes to GP, inaccessible mental health services, denial by daughters. Minority ethnic groups: fear of negative judgements and gossiping.	Good and trustful bond with daughters, good contact with school	Medium
Flink, 2013b, The Netherlands	Qualitative	*n* = 50	12–20-year-old female adolescents from different ethnic backgrounds	Negative attitudes towards health professionals and school services. Minority groups: fear to parental and fear reactions	Not assessed	Medium
Gonzlaves, 2012, Portugal	Qualitative	n = 39	12–17 years old immigrant attending to school, parents, teachers and health professional.	Adolescents: reliance on self-support, shyness, fear and language gap Family: fees, language, legal issues Professionals: non-recognition of the problem All: stigma	All: strong link with community, mainly teachers and health professionals	Medium
Gulliver, 2012, Australia	Qualitative	n = 15	16–23-year-old elite athletes (66.7% aged under 19).	Stigma, lack of mental health literacy, negative past experiences of help-seeking	Encouragement from others, stablished relationship with provider, previous positive experiences with mental health services, positive attitudes of others, access to internet.	Medium
Haavik, 2017, Norway	Cross sectional	*n* = 1249	Adolescents from Norwegian upper schools (mean = 17.6)	Mental health literacy, delay in making contact, stigma.	Increased mental health literacy, awareness of service availability	Medium
Hasset, 2017, United Kingdom	Qualitative	n = 8	16–18-year-old males who entered CAMHS following self-harm or suicidal ideation and where engaged in therapy	Want to maintain an independent self.	External adult recognising, normalising and initiating help-seeking. Greater insight, maintaining independent self.	High
Hernan, 2010, Australia	Cross sectional	*n* = 74	14 to 16 years old high school students from rural and metropolitan towns	Personal factors related with communication with mental health professionals, problem recognition, shame, confidentiality breach. Logistical factors (transport, money, travel distances, etc.).	Not assessed	Low
Ijadi-Maghsoodi, 2018, United States	Qualitative	*N* = 76	11–18 years old school students	Embarrassment, fear of judgement, confidentiality, mental health literacy		Medium
Jennings, 2015, United States	Cross sectional	*n* = 246	18–24 (73.3% aged 18–19) college students	Perceived stigma, self-stigma, higher self-reliance	Not assessed	Low
Kahi, 2012, Lebanon	Cross sectional	*n* = 521	17–21 years old student (53,8% aged 17–18) undergoing a preventive medical visit at University centre	Confidentiality, embarrassment, doubt about the professionals’ ability to act, knowledge of services, and logistical factors (money, transport, contact).	Not assessed	Low
Labouliere, 2015, United States	Cross sectional	*n* = 2145	14–18-year-old high school students	Extreme self-reliance	Not assessed	Low
Linsdey, 2010, United States	Mixed-method	*n* = 69	13 to 18 years old African American boys with high levels of depressive symptoms	Shame and distrust of mental health professionals	Not assessed	Medium
Lubman, 2017, Australia	Cross sectional	*n* = 2456	14–15-year-old high school students	Self-reliance, embarrassment, time and money	Not assessed	Medium
Lynn, 2014, United Kingdom	Cross sectional	*n* = 175 adolescents *n* = 95 parents	14–18-year-old adolescent	Adolescents: desire of being independent, reduced mental health literacy in parents.	Adolescents: Higher perception of problem severityBoth: prior professional help-seeking.	Low
Maioulo, 2019, Australia	Cross sectional	*n* = 1582	16–18 years high school students	Not assessed	Positive parenting	Medium
Mariu, 2012, New Zealand	Cross sectional	*n* = 9699	12–18 years old secondary students (years 9 and 10).	Not assessed	Living with a single parent, living in an over-crowed house, being well known by a teacher	Medium
Maritnez-Hernaes, 2014, Spain	Cross sectional	*n* = 105	17–21 year old (84.3% aged under 19) participating in longitudinal survey	Normalisation of problem, stigma, reliance on self, beliefs of no need of professional help	Positive perception of mental health professionals	Medium
McLean, 2013, United Kingdom	Qualitative	*n* = 90	10–15-year-old secondary school students	Stigma	Not assessed	Medium
Murry, 2011, United States	Mixed Methods	*n* = 163	African American mothers of adolescents (mean = 14) living in rural Georgia	Community stigma towards family, cultural mistrust, cost	Welcoming environment of mental health services	Medium
Nearchou, 2018, Ireland	Cross sectional	*N* = 722	12–16 years old school student	Perceived public stigma	Not assessed	Medium
O’Connor, 2014, Australia	Cross sectional	*n* = 180	17–25-year-old (74.16% aged 18–19) college students	Not assessed	Extraversion, increased perceived benefits of help-seek, low social support and high perceived benefit	Low
Pisani, 2012, United States	Cross sectional	*n* = 2737	14–17 years old high school students in rural communities	Not assessed	Positive attitudes about help-seeking, perceptions responsiveness from adults, school support	Medium
Recto, 2018, United States	Qualitative	n = 20	15–19 years adolescents with perinatal depression	Fear of judgement, normalisation of symptoms, lack of trust	Not assessed	Medium
Rughani, 2011, Australia	Cross sectional	*n* = 778	13–18 years old high school students (years 9 to 12) in rural towns	Mistrust and do not believe professional help is beneficial	Perceived benefits of mental health treatments	Medium
Samuel, 2014, United States	Qualitative	n = 54	15–17-year-old African American males who received mental health treatment services after detention	Stigma, ineffective treatment, fear and shame from peers, mistrust of mental health providers	Not assessed	Medium
Sawyer, 2011, Australia	Cross sectional	*n* = 5362	12–14 years old school students	Higher depressive symptoms	None found	Medium
Seamark, 2018, United Kingdom	Qualitative	n = 6	17–18-year-old college psychology students	Gender roles, cultural expectations, lack of awareness of sources of help, fear of stigma and rejection	Not assessed	High
Sharma, 2017, India	Cross sectional	*n* = 354	13–17-year-old school students	Feeling ashamed, uncomfortable	Not assessed	Low
Shechtman, 2018, Israel	Cross sectional	*n* = 238	14–18-year-old school students	Self-stigma	Not assessed	Low
Sylwestrzak, 2015, United States	Cross sectional	n = 10,123	13–18-year-old adolescents	Self-reliance, mental health literacy, fear of stigma, usefulness of treatment	Not assessed	Low
Tharaldsen, 2017, Norway	Qualitative	n = 8	17–18-year-old students	Limited knowledge, stigma	Not assessed	Medium
Thomas, 2013, Australia	Cross sectional	*n* = 289	18–25-year-old (59.9% aged 18–19) students enrolled in first year psychology classes	Not assessed	Good symptom recognition, identification of benefits of professional help, openness to treatment for emotional problems	Medium
Wang, 2018, United States	Mixed methods	n = 19	Asian immigrants parents	Mental health literacy, structural barriers, cultural barriers (stigma, lack of cultural sensitivity of services)	Not assessed	Medium
Watsford, 2014, Australia	Cross sectional	*n* = 102	12–18-year-old presenting mild to moderate mental health concerns	Not assessed	Previous experience in mental health services	Medium
Wilson, 2010a, Australia	Cross sectional	*n* = 109	18–25 years old (78% age 18–19) college students*	Higher levels of psychological distress, negative beliefs about treatment	Not assessed	Medium
Wilson, 2010b, Australia	Cross sectional	*n* = 302	18–25 years old (78% age 18–19) university students*	Higher level of suicidal ideation and higher depressive symptoms	Not assessed	Medium
Wilson, 2010c, Australia	Cross sectional	*n* = 590	13–18 years old high school students	Higher levels of suicidal ideation and general psychological distress.	Not assessed	Medium
Wilson, 2011, Australia	Cross sectional	*n* = 562	18–25 years old (87,7% aged 17–21) students	Need for autonomy and independence	Not assessed	Medium
Wilson, 2012, Australia	Cross sectional	*n* = 1037	13–21-year-old adolescents (79% under 19)	Need for autonomy	Helpfulness of prior mental health care	Low
Yap, 2011, Australia	Cross sectional	*n* = 3746 teens *n* = 2005 parents	12–25 years old student (separated data)	Stigma and beliefs about helpfulness of mental health care	Not assessed	Low
Yoshioika, 2015, Japan	Cross sectional	*n* = 311	15–19 high school students	Concerns about what other people may think	Not assessed	Low
Zhao, 2015, Canada	Cross sectional	*N* = 115	15–16-year-old students	Not assessed	Secure attachment style, strong relation with peers	Medium

The majority of studies were conducted in educational settings, such as schools (*n* = 24) and tertiary education (*n* = 11) focusing in non-clinical samples. Sixteen studies included participants from other community settings and two studies were conducted in mental health care facilities. Among the studies that include actual help-seekers (*n* = 7), the most common reason for seeking help was suicidal ideation, self-harm, depressive symptoms, and general mental health concerns (e.g., anxiety/nervousness/fear). Therefore, the conclusions drawn by the majority of the articles were based on help-seeking intentions rather than actual behaviours, since the participants were not experiencing mental health problems and focused on hypothetical scenarios.

### Help-seeking barriers

#### Stigma

Stigma is defined as the fear of being socially sanctioned or disgraced leading to hiding or preventing certain actions or behaviours, including the misreporting of mental health problems [81]. More than half of the included studies (*n* = 30) made reference to this and other negative attitudes towards mental health problems as the main obstacle to help-seeking behaviours in adolescents. Of these, twenty-five studies referred to stigma as the primary obstacle, describing it through different concepts such as, “stigma”, “fear of stigmatisation”, “community stigma”, “perceived stigma” and “self-stigma”. Other negative attitudes towards mental health problems included shame, fear, and embarrassment.

#### Family beliefs

The second most mentioned barrier was associated to adolescents’ family beliefs toward mental health services and treatment (*n* = 15). Barriers related to problem with communication and distrust towards health professionals, negative past experiences with mental health services, and believing that the treatment is not going to be helpful. This was especially true for studies including immigrant and refugee populations, which referred to cultural barriers including mistrust of mental health diagnosis and practitioners, and lack of cultural sensitivity in services as a significant barrier.

#### Mental health literacy

Mental health literacy refers to the ability to use mental health information to recognise, manage and prevent mental health disorders and make informed decisions about help-seeking and professional support [82]. Almost one-third of the articles (*n* = 14) referred to problems related to mental health literacy as a significant barrier including poor recognition of mental health conditions (self and others) and lack of awareness of available sources of help.

#### Autonomy

Adolescents’ attitudes towards help-seeking revealed a perceived need of self-sufficiency and autonomy which were recognised as a relevant barrier in twelve studies, as well as fears of confidentiality breaches.

#### Other help-seeking barriers

To a lesser extent, problems regarding service and personnel availability and other structural factors (such as cost, transportation and waiting times) were mentioned as obstacles to help-seeking (*n* = 8). This was a significant barrier for studies including rural and immigrant populations, and in studies that included parents in their sample. Six studies focused on the relationship between symptomatology and help-seeking. These found that higher levels of psychological distress, suicidal ideation and depressive symptoms were linked to lower help-seeking behaviours.

### Help-seeking facilitators

Of the 56 included studies, 19 also referred to facilitators of help-seeking behaviours. Mental health literacy and prior mental health care were the most cited facilitators for help-seeking for mental health problems (*n* = 10). Specifically, timely access to mental health was facilitated by having a previous positive experience with mental health services or help-seeking, being familiar with the sources of help, and good symptom and problem recognition. Higher engagement with the community and having a trusting and committed relationship with relevant adults such as parents, schoolteachers and counsellors also facilitated seeking help among adolescents. Further details of the included articles are available in Table 2.

### Secondary outcomes

Few studies identified a significant difference when comparing younger and older adolescents in relation to barriers and facilitators to help-seeking, with no conclusive findings being reached. Some findings suggested that older adolescents tended to establish to feel more comfortable with people with mental health issues [83] and had less help-seeking fears [40]. In contrast, younger adolescents had greater knowledge about professional sources of help [34]. Only one study found a significant difference between ages regarding help-seeking, with younger adolescents reporting higher intentions of seeking help [60].

Twenty-four studies examined possible gender differences in help-seeking barriers and facilitators. Seven studies did not find significant differences between genders [28, 39, 40, 42, 46, 51, 69]. One study reported higher help-seeking intentions in males experiencing suicidal intentions [60] and two studies found that females perceived more overall barriers [26–58]. However, this may be related to higher rates of females seeking help for mental health problems compared to males [31, 33, 37, 42, 48, 53, 58, 61, 76]. Studies reviewed did not evidence convincing differences between gender in relation to help-seeking.

### Question 2: help-seeking interventions

Thirty-six studies on interventions targeting help-seeking behaviour, including a total of 28,608 participants, were summarised in the review (Table 3). Most of intervention studies were conducted in Australia (14) and the United States (14), followed by Canada (4) and United Kingdom (3). All studies were conducted in educational setting including high school (*n* = 35) and college (*n* = 1). The majority of studies developed interventions for non-clinical samples, and their focus was the prevention of mental health problems and the promotion of healthy coping strategies via help-seeking behaviours. Outcomes varied between help-seeking intentions, attitudes and behaviours. Almost half of the studies focused on the effectiveness of the interventions, while sixteen were feasibility or pilot trials and study protocols. Most of the studies used a quasi-experimental design (*n* = 21) followed by randomised controlled trials (*n* = 15). The age of participants ranged from 11 to 19 years old, although one study that included participants under 29 years old was incorporated as more than half of the sample were adolescents. Interventions were delivered using four main methods: psychoeducation, outreach interventions, multimedia tools and peer leader training.

**Table 3:** Help-seeking interventions

1st Author, year, country	Sample size	Inclusion criteria	Design	Setting and intervention	Comparison group	Primary outcome	Measures	Results and effect size	Quality
Psychoeducation-mental health									
**Berridge, 2011, Australia**	182	10th grade students (aged 14–16)	Post-test design Feasibility trial	MAKINGthe LINK. School-based. Five class room activities provided by teachers who received specialised training.	No control group	Acceptability and feasibility of the programme	A 16-item programme satisfaction survey.	-Not relevant	n/a^1^
**Casañas, 2019, Spain**	.	13–14 year old high school student	Clustered RCT^2^. Study protocol	EspaiJOVE, 14 h of psychoeducation and activities with the scholar community.	Waiting list	Help seeking behaviours	General Help-Seeking Questionnaire (GHSQ)	-Not relevant	n/a
**Lubman, 2016, Australia**	–	year-9 students (aged 14–15)	Cluster RCT Study protocol	MAKINGthe LINK. Five class room activities provided by teachers who received a training.	Waiting list	Help seeking behaviours	General Help Seeking Questionnaire (GSHQ-V)	-Not assessed	n/a
**Perry, 2014, Australia**	208	9th or 10th grade students (aged 13–16)	Cluster RCT. Feasibility trial	HeadStrong, school based intervention with educational practical modules during 5 to 8 weeks.	Regular Health and Physical Education classes	Attitudes towards help-seeking	Inventory of Attitudes towards Seeking Mental Health Services (IASMHS)	-Not relevant	Medium
**Sharpe, 2016, United Kingdom**	6551	Year 7 school student (aged 11–12)	Hierarchical cluster RCT.	Student booklet designed fomenting help-seeking and self-management support.	Waiting list	Help-seeking behaviour	Four-point scale developed by authors	There was no difference in help-seeking behaviour Odds ratio (95% C.I) 1.01 (0.95–1.07)	Medium
Psychoeducation-depression									
Joyce, 2011, Australia	32	Secondary students (aged 14–16)	Post-intervention design. Pilot study	School based. Information sheet based on evolutionary perspective	Informative sheet about depression	Piloting the sheet.	Linkert scale (5 points)	-Not relevant	n/a
King, 2011, United States.	416	9th to 12th grade high school students	Quasi experimental design	Surviving the Teens is school based programme. Four 50 min session focused on psychoeducation.	No control group	Help seeking attitudes and behaviour	Instrument developed for the study evaluating self-efficacy and help-seeking behaviours.	Increased help-seeking behaviours after intervention and maintained in follow-up (3 months). (*t* = 4.634/ *p* < .001)	Medium
Robinson, 2010, Australia	246	Boys school students (aged 14–16)	Pre-test, post-test design	School-based developed in one-off, 2-h workshop focused on depression definition, coping skills and help-seeking.	Waiting list	Help-seeking behaviours	Evaluation of Mental Health First Aid training	Intervention group was more likely to seek for help Adjusted odds ratio (95% C.I.)= 3.48 (1.93, 6.29) *p* < 0.0001	Medium
Ruble, 2013, United States	593	High school students	Pre-test and post-test design	School-based, 3 h curriculum designed to educate students, teachers, and parents about depression and help-seeking.	No intervention	Attitudes towards treatment seeking	Adolescent Depression Knowledge Questionnaire (ADKQ)	Significant increase in the intention to seek help from other *t* = 13.658/ *p* < 0.0001.	Medium
Strunk, 2014, United States	158	Emotionally troubled teens in 9th and 12th grade	Pre- test and Post-test design	Surviving the Teens is school based programme. Four 50 min session focused on psychoeducation.	No control group	Help-seeking behaviours	Survey developed by authors.	Paired t-test showed a significant increase at posttest (*p* < 0.0005) but not at 3 months follow-up (*p* = 0.014).	Medium
Beaudry, 2019, United States	481	High school students	RCT.	Interactive classroom curriculum and focus group with parent, students and teachers.	Waiting list	Help seeking behaviours related to depression	Adolescent Depression Knowledge Questionnaire (ADKQ)	-Not available due to data collection issues	Medium
Howard, 2019, Australia	327	16–19 year old students	Three-arm, pre-posttest, double-blind RCT.	Psychoeducation of biological and psychosocial conditions and causes of depression	Neutral information about depression	Help-seeking intentions	General Help-Seeking Questionnaire (GHSQ)	-Small but significant increase in help-seeking (*p* = 0.015)	Medium
Psychoeducation-stigma									
Calear, 2017, Australia	–	11 and 12 grade male students (15–18 years of age)	Two arm cluster RCT.Study protocol	Silence is Deadly. School based, focused in males. Included classroom presentation (1 h), and supporting website and social media messages.	Waiting list	Help seeking intentions	General Help-Seeking Questionnaire (GHSQ)	-Not assessed	n/a
Hart, 2016, Australia	241	High school students (aged 15–17)	Pre-test, post-test design Feasibility trial	Teen Mental Health First Aid program. School based, 3 sessions, delivered to students, parents and teachers.	No control group	Help seeking intentions	Survey questionnaire developed for the study to measure help-seeking.	-Not relevant	Medium
Rickwood, 2004, Australia	457	School students (aged 14–18)	Solomon four-group design (pre-and post-intervention).	School based, interactive presentation by former mental health patients.	No intervention	Help-seeking intentions	General Intentions to Seek Help Questionnaire	Significant effect of the intervention Wilks’ Λ = .942 F (4,417) = 6.428*p* < 0.001	Medium
Saporito, 2013, United States	156	Adolescents from Public High School	RCT.	School based Interactive 1 h session, providing basic mental health and video with case example.	Educational presentation with content unrelated to mental health.	Reduction of Stigma towards help-seeking	Short for of Attitudes toward Seeking Professional Psychological Help (ATSPPH)	Increased willingness to seek for help in students with past treatment history F(1, 146) =6.64, *p* = .01ηp2 = .04	Medium
12. Yang, 2018, United States	14	High school students “at risk” of mental health conditions.	Quasi-experimental Pilot study	InSciEd-oOut. School based, 20-day anti-stigma classroom activities	No control group	Help-seeking behavioural intentions	General Help-Seeking Questionnaire Vignette Version.	Not assessed	n/a
30. Young, 2013, Canada	254	High school students (aged 14–18)	Pre and Post intervention survey design	School based, combining classroom sessions and talk with person with schizophrenia	No control group	Self-stigma in help-seeking	Self-Stigma of Seeking Help scale	Significant reduction in self-stigma to seeking help after the intervention (*p* < 0.05)	Medium
Psychoeducation- suicide + self-harm									
Aseltine, 2004, United States	2100	Students in high schools	Cluster RCT	SOS programme, school based interactive intervention based on teaching materials and discussion guide, plus a screening instrument.	Health and social studies classes	Help-seeking behaviour	Questionnaire developed by the authors	Not significant effect of the program in help-seeking β = 0.255 *p* > 0.05	Medium
Aseltine, 2007, United States	3837	Students in high schools in Hartford and in Columbus.	Cluster RCT	SOS programme, school based interactive intervention based on teaching materials and discussion guide, plus a screening instrument.	Health and social studies classes	Help-seeking behaviour	Questionnaire developed by the authors	Not significant effect of the program in help-seeking β = −0.01 *p* > 0.05	Medium
Freedenthal, 2010, United States.	142 staff 146 student	Staff and students in High school in Denver area.	Pre and Post intervention survey Pilot study	Yellow-ribbon. School based, 25 min training, card with hotline numbers and t-shirt with mental health messages.	No intervention	Help seeking behaviours	Self-reported survey developed by authors	Not relevant	n/a
Kalafat, 1994, United States	253	10th grade high school students	Solomon four-group design	School based suicide awareness program. 3 psychoeducation sessions.	Physical education classes	Help seeking attitudes	Likert scale developed by the authors.	Significant effect of the intervention F (14,225) =1.87 *p* < .03	Medium
Schimidt, 2015, United States	5949	High school students (aged 10–18)	Pre and Post intervention design. Feasibility trial	Yellow-ribbon. School based, 25 min training, card with hotline numbers and t-shirt with mental health messages.	No control group	Help Seeking	9 item scale developed for the study	-Not relevant	n/a
Outreach interventions									
Deane, 2007, Australia	506	9th and 10th grade high school students	Post-test design	The Building Bridges to General Practice, school based. GP deliver one session to improve students perception.	No intervention	Help seeking intentions	Adapted version from the General Help Seeking Questionnaire (GHSQ)	Increased help-seeking intentions at follow-up *F*(2,217) = 3.04/ *p* < 0.05.	Medium
Rughani, 2011, Australia	260	Year 11 high school students	RCT	Promoting Access to support seeking. School based, two 50 min sessions for building relationships with mental health professionals.	Alternative presentation	Help-seeking intentions	Items adapted from the General Help-Seeking questionnaire (GHSQ)	Short term improvement of help-seeking intentions F (14,225) =1.87 *p* < .03	Medium
Wilson, 2008, Australia	291	Year 11 students from 3 high schools in New South Wales	Quasi-experimental Nested design	The Building Bridges to General Practice, school based. GP deliver one session to improve students perception.	No intervention (year 10 students)	Help-seeking intentions	General Help Seeking Questionnaire (GHSQ)	Increased help-seeking intentions for psychological problems after the intervention F(2,598) = 4.31*p* < 0.01	High
Multimedia/Online interventions									
Conrad, 2014, Germany	532	High school Students without history of mental health care	Quasi-experimental	Film festival aiming to give a podium to the topic mental health.	No control group	Help seeking attitudes	Seven items Linkert scale developed by authors	Not significant change in help-seeking *t* = 0.414	Medium
Nicholas, 2004, Australia	243	High school students aged 13 to 18.	Post-test design Feasibility trial	School based, classroom presentations of ReachOUT! Website.	No control group	Help-seeking behavioursintentions and the use if the website.	Questionnaire developed by authors	Not relevant	n/a
Rowe, 2018, United Kingdom	23	Teens (aged 12–18) who had self-armed (last 12 months) and basic English speaker.	Two group parallel arm, single blind RCT.Feasibility trial.	My self-help tool. School based, web-personalised decision aid intervention designed to help identify help-seeking alternatives for self-harm.	General information about mood and feelings.	Help seeking intentions	General Help-Seeking Questionnaire (GHSQ).	Not relevant	n/a
Santor, 2007a, Canada	388	Grade 8th high school students	Pre-post test design	School based help seeking workshop + Information website called “YooMagazine”	No intervention	Help-seeking attitudes	The Help Seeking Attitude Questionnaire	Positive effect of the intervention β = 0.108, F(1,1037) = 3.85 *p* < 0.04.	Medium
Santor, 2007b, Canada	455	Grade 7th to 12th students	Pre and Post intervention survey design Feasibility trial	Information website called “YooMagazine”	No control group	Help-seeking behaviours	Help-seeking indicators (e.g: visits to school mental health)	Not relevant	n/a
Wiljer, 2016, Canada.	–	Youth (16–29) college students	Two arm RCT.Study protocol	Thought Spot. College based, web and mobile platform	Usual care	Self-efficacy for mental health help-seeking	General Help-Seeking Questionnaire (GHSQ)	Not assessed	n/a
Peer leader training interventions									
Calear, 2016, Australia	–	7 to 10 grade students (aged 12–15)	Two arm cluster RCT. Study protocol	The sources of Strength. School based, during 1 year (approx.) includes staff and peer leaders training and school messaging.	Waiting list (24 months).	Help seeking intentions	An adapted version of General Help -Seeking Questionnaire(GHSQ)	Not relevant	n/a
O’reilly, 2016, Ireland	30	Teens aged 15–17 with interest in mental health, speaking skills.	Pre and Post intervention survey design Feasibility trial	School based, four 3 h workshop training peer leaders.	No control group	Stigma for help-seeking behaviours	Perception of Stigmatisation by Others FOR Seeking Help Scale (PSOSH)	Not relevant	n/a
Parihk, 2018, United States	878	High school students	Quasi-experiment	P2P. School based, peer leaders develop a school public awareness campaign	No control group	Help seeking intentions	Adapted version of the P2P Depression Awareness Questionnaire	78% of students will seek for help after the intervention *p* < 0.001	Medium
Wyman, 2010, United States	2675	High school students (aged 14 to 18)	RCT.	The sources of Strength. School based, during 1 year (approx.) includes staff and peer leaders training and school awareness campaign.	Waiting list	Acceptability of seeking help	Help Seeking from Adults scale created by authors	Positive effects on help-seeking from adults Mean differences (95%CI) =0.58 (0.24, 0.91). *p* = 0.04	Medium

^1^ n/a: Not applicable

### Types of intervention

#### Psychoeducation

Most of studies (*n* = 23) used psychoeducation and classroom-based interventions. Although all the interventions focused on encouraging help-seeking behaviours, the emphasis and content differed among them, including general mental health topics, suicide and depression awareness and stigma.

Five studies developed programmes based on the notion that promoting mental health awareness could enhance mental health literacy and promote help-seeking [84–88]. Four interventions targeting help-seeking for suicide were identified within five studies [89–93]. Five interventions explicitly targeted help-seeking for depression in school-based settings their focus being to educate the school population about adolescent depression and thereby encourage help-seeking [94–98]. Two studies evaluated the effectiveness of an intervention combining depression awareness and a suicide prevention programme promoting early identification and self-referral [99, 100]. Six classroom-based interventions addressing stigma were identified, two of which used psychoeducation to overcome myths regarding mental illness [101, 102] and four focused on providing interpersonal contact with people with mental health conditions in order to improve acceptance and increase help-seeking intentions [103–106].

#### Outreach interventions

Three studies used outreach interventions to target mental health help-seeking [107–109]. These aim to establish contact with adolescents who may be experiencing psychological and emotional distress in order to help them get the attention they need and increase their access to health services. They were based on the Building the Bridges to General Practice (BBGP) programme, developed by Wilson et al. (2005), a programme that aims to target help-seeking obstacles for physical and psychological problems by promoting contact between high school students and general practitioners [110].

#### Multimedia interventions

Six types of multimedia interventions have been developed to address some of the difficulties of reaching an adolescent population, such as fear of confidentiality breaches, stigma and self-reliance [111–115]. The interventions included interactive films to engage students with mental health related topic and online platforms providing personalised information regarding the decision-aids process.

#### Peer training interventions

Peer training interventions are focused on the training of peers who act as active agents of change and social interactions incorporated into the daily activities within the school environment [116]. All three programmes followed similar principles concerning improving the climate around mental health problems, promoting social connectedness, and challenging norms and behaviours associated with help-seeking [117–120]. “Peer leaders” acted as a link between the student population and mental health literacy, promoting the acceptability of seeking for help for mental health problems.

Further details of the included articles are available in Table 3.

### Secondary outcomes

No studies referred to significant differences concerning the effectiveness of help-seeking interventions when comparing ages. No significant gender differences were identified regarding the effectiveness of the help-seeking interventions [89, 101, 103, 111]. However, before the intervention¸ females tended to have higher mental health literacy and more adaptive attitudes regarding mental health problems [90, 111], including greater help-seeking knowledge and intentions [107, 112, 113].

### Effectiveness

The main goal of this review was to describe the interventions targeting help-seeking in adolescents and therefore did not include an analysis of their effectiveness. Almost half of the included studies were study protocols and feasibility studies, so effect sizes were not reported. However, some findings are worth mentioning.

Four studies which looked at effectiveness of the interventions focused on psychoeducation about depression found a significant effect in increasing help-seeking. King et al., [99] identified that there was an increase in future help-seeking behaviours after the interventions and that this was maintained at 3 months’ follow-up (*t* = 4.634/ *p* < .001). Strunk et al., [100] found a significant increase of help-seeking (*p* < 0.0005); however, this was not sustained at follow-up (*p* = 0.014). Robinson et al., [95] found that the intervention group was more likely to seek help at post-test (Odds ratio (95% C.I) =3.48 (1.93, 6.29), *p* < 0.0001) and Ruble et al., [96] found increased intention of help-seeking from others after the intervention (*t* = 13.658/ *p* < 0.0001.).

The three studies that looked at the effectiveness of stigma reduction identified positive effects of the intervention on help-seeking. Two studies [101, 104] found a significant reduction in self-stigma surrounding seeking help after the intervention (*p* < 0.05) and one study [103] found a significant effect of the intervention in help-seeking intentions (Wilks’ Λ = .942, *F* (4,417) = 6.428, *p* < 0.001).

Finally, all the studies that focused on outreach found a significant effect of the intervention in help-seeking intentions. One detected an increase in intentions at 3 months follow-up (*F* (2,217) = 3.04/ *p* < 0.05) [108], Rughani [107] found short terms improvements in help-seeking intentions (F (14,225) =1.87 *p* < .03) and Wilson [109] found a significant effect in the intention of seeking help for psychological problems after the intervention (F (2,598) = 4.31 *p* < 0.01).

### Quality assessment

The majority of the studies were low to medium quality with moderate to high risk of bias. Most of the cross-sectional studies did not state a clear inclusion and exclusion criteria and did not consider possible confounders affecting the interpretation of the outcome. Regarding qualitative research, the most common problem was linked to sample size and the difficulty of providing a clear strategy to address the subjectivity of the authors in the interpretations of the data. Mixed method studies presented some inconsistencies in addressing specific components of both quantitative and qualitative traditions, and in the process of integrating both approaches. Regarding intervention studies, it was difficult to identify to what extent the groups were similar at baseline. Although some studies included baseline measures of demographic information, most of them did not consider confounders or other factors influencing effectiveness, and some studies did not have any baseline measures. Also, few studies included follow-up and the ones that did, had high attrition rates and short follow-up periods (up to 6 months); therefore, it is not possible to attribute a long-lasting effect to the interventions. Quasi-experimental studies acknowledge possible selection and sample bias. Randomised controlled trials presented difficulties in terms of the blinding of the research team and participants at different stages of the process.

Overall there was inconsistency regarding the measurements of help-seeking, with most of the studies focusing on help-seeking intentions, which is not necessarily related to future behaviours. Moreover, many studies did not use valid and reliable instruments for measuring help-seeking. This is especially true for the experimental studies since most of them developed tools focused on their intervention rather than standardised help-seeking measures. Finally, most of the studies only used self-report measures, increasing the risk of bias of the findings. We did not assess the quality of study protocol, feasibility studies and pilot studies.

## Discussion

### Question 1: barriers and facilitators

This review focused on identifying barriers, facilitators and interventions targeting help-seeking behaviours in adolescents. Consistent with previous findings [1], the most prominent barrier identified was stigma. Negative attitudes and beliefs about mental health services and professionals was the second most prominent barrier. Trusted and strong relationships with possible gatekeepers (teachers, parents, GPs, health professionals, etc.) and prior positive help-seeking experience were the most cited facilitators.

Few studies related symptom severity with help-seeking. Of those that did, higher symptomatology was associated with lower help-seeking intentions and behaviours. This is in line with previous studies suggesting that teens who are most in need are less likely to seek help [1, 11, 15]. It is possible that the nature of mental health symptoms such as self-blame, emotional distress, difficulty in speaking to others and diminished cognitive ability contribute to lower help-seeking behaviours. Adolescents with higher symptom severity may be even more vulnerable experiencing difficulties with the help-seeking process in areas such as identifying the need for professional assistance or fear of stigmatisation. This could be due to higher rates of isolation and exclusion from their peers. Increasing mental health literacy among this population may provide a way of improving social support between peers [121].

There are structural barriers affecting the help-seeking process that go beyond attitudes, for example, costs, waiting times and transportation. These barriers were not among the most prominent reasons cited in the research review; however, this may be related to the limited amount of studies that included parents’ perceptions. A previous review, which focused on the parents of children and adolescents, concluded that structural barriers were the most relevant [122]. This suggests that adolescents are less worried about the practical implications of accessing help for mental health problems and are more affected by being attitudinal barriers, but that structural barriers may be more relevant to parents.

Key facilitators to help-seeking should be considered when creating new interventions such as trusted relationships with gatekeepers, and familiarisation with the help-seeking process. However, the lack of studies focusing on facilitators precludes many conclusions being drawn. The majority of studies used sub-clinical samples and/or hypothetical help-seeking scenarios rather than asking genuine help-seeker with mental health problems who could refer to the real circumstances leading them to ask for help. More research including young people who have sought help from services would be useful in understanding the idiosyncrasies of this process.

These findings provide a useful overall picture of the relevant factors influencing the help-seeking process in adolescents. However, the included studies did not share a clear definition and framework regarding help-seeking. A wide range of tools were used to measure help-seeking, varying in their validity and reliability, and also in the constructs they measured. This limits the generalisability of the findings and our understanding of the help-seeking process. Rickwood & Thomas (2012) have proposed a framework regarding help-seeking, identifying the different parts of the process, sources of help, types of help and main concerns [15]. In the future, sharing such a framework could be a useful means to reach a general agreement regarding the definition of help-seeking and its components.

### Question 2: interventions

The types of interventions varied considerably and included classroom-based psychoeducation, outreach interventions, multimedia and online-based interventions and peer training. Among classroom-based psychoeducation interventions, the most effective ones were those focused on prompting help-seeking through addressing depression and stigma. All peer outreach interventions had a significant effect in improving help-seeking intentions, thus showing promising results. In sum, addressing stigma, mental health literacy, and attitudes towards mental health services could be beneficial in terms of promoting help-seeking.

Most of the intervention studies included in this review did not investigate mechanisms of change with regards to help-seeking behaviour. The relevance of studying underlying mechanisms and practical requirements related to the functionality of interventions has been previously discussed [123], and most of the interventions included in this review did not refer to these processes. Identifying such mechanisms could help understand how interventions work, enlightening and optimising the process of decision-making and design [93]. Adolescence is a period essentially characterised by emotional, behavioural, hormonal, and neuronal changes [124, 125]. Interventions congruent with the developmental stages may be useful to target age-appropriate factors.

It is important to mention that few intervention studies referred in detail to the implementation process and the main issues they encountered; however, the studies which did refer to this, found significant barriers. School administration issues, the difficulty of obtaining parental consent and attrition rates for the follow-up measures were one of the main difficulties regarding the implementation [93, 97, 102, 115]. Teacher’s support and engagement with the intervention were also described as a barrier in the implementation process for some studies [85, 93, 114]. Most of studies concluded that implementation strategies should consider the reality and challenges of each school. For this the theme of contextualization is fundamental and the specificities of the process of implementation (planning, engaging, executing, reflecting and evaluating) [126, 127].

All interventions were conducted within an educational setting. Special attention should also be paid to young people outside of the educational system, who are particularly vulnerable in terms of economic and social deprivation [128]. Around one in five children and adolescents are out of school according to the UNESCO [129], with psychosocial factors appearing to obstruct traditional educational trajectories [130]. Health and mental health conditions have a relevant role in terms of absenteeism and truancy [131]. Adolescents experiencing symptoms of depression and anxiety or in charge of a chronically sick relative can be more prone to avoid school and stay at home. These children can be even more vulnerable and harder to reach, and there is a lack of collaborative effort attempting to overcome this situation. Encouraging partnerships between the health and educational systems, community settings, youth detention centres, among other institutions providing social care, should be promoted with the purpose of supporting mental healthcare and provision for young people [132].

Encouraging adolescents to seek help for mental health problems is a key priority however, this does not resolve the discrepancy between needs and resources worldwide [132, 133]. “Mental health services for children and adolescents have internationally been poorly understood, underfunded and even neglected by governments” [p.92, 134]. This may be associated with the lack of a general understanding of this population’s needs (including developmental issues), and the “implementation gap”, referring to the challenges of translating evidence to health service development and practice [134]. Simultaneously, focusing on increasing help-seeking and service availability for children and adolescents is necessary to reduce the global burden of disease and protect the future health of this population [125, 135].

### Limitations

This review has a number of limitations. First, only one author performed the data extraction and critical appraisal of papers therefore the data analysis is at risk of some subjectivity. Second, there is an increasing debate regarding the age that adolescence comprises, with some suggesting the age should be extended to 10 to 24 years old [17]. However, we decided to follow the definition of ´adolescent´ established by international organisations including the OMS and UNICEF. A significant number of papers were excluded considering our age range (*n* = 104). Defining adolescence as a period between 10 to 19 years old could be a limitation to our study. Thirdly, this review focused on common mental health problems such as depression, anxiety and emotional distress and excluded psychiatric conditions such as anorexia, schizophrenia and substance misuse, mainly due to the particular nature of the help-seeking processes. However, the exclusion of substance misuse problems could be seen as a limitation of this study due to its high prevalence in adolescence, making it a particularly sensitive issue during this period of life [136]. Finally, this review prioritised the overinclusion of studies to have an overall picture of the existing evidence regarding help-seeking for mental health problems in adolescents. As a result, low quality studies were included in the analysis and may affect the interpretation of the findings. There were some notable strengths in this review. This is the first systematic review studying help-seeking barriers, facilitators and interventions in order to give a comprehensive review of the topic. The search strategy developed was over-inclusive, using an optimal database combination, including multiple languages and PPI involvement in the development of the topic.

## Conclusion

In conclusion, stigma and negative beliefs about mental health services appear as the most significant barriers to help-seeking for adolescents, whereas previous positive experiences with services and good mental health literacy are the most relevant facilitators. There are a number of interventions being developed to promote help-seeking for mental health problems in adolescents, and most of them take place in high education settings. They include a range of delivery methods including psychoeducation, stigma and depression awareness campaigns, online tools and peer training. Since such initiatives are relatively new, there is a need for more trials, with longer follow-up periods and the use of reliable and validated tools focused in future help-seeking behaviour. Despite school seeming to be the ideal setting for deploying these interventions, it is important to consider adolescents outside the school system who may be in more need of attention for psychosocial and mental health problems.

## Supplementary information

**Additional file 1.**

## Data Availability

Data sharing is not applicable to this article as no datasets were generated or analysed during the current study.

## Abbreviations

MMAT: Mixed Methods Appraisal Tool
RCT: Randomised Controlled Trial
